# Nomogram based on tumor burden score for prediction of prognosis of patients with hepatocellular carcinoma before hepatectomy

**DOI:** 10.3389/fonc.2025.1578859

**Published:** 2025-07-08

**Authors:** Qianru Xiao, Zhengqing Lei, Anfeng Si, Xuewu Tang, Facai Yang, Weihu Ma, Cheng Chi, Qiushi Yu, Yigang He, Haolan Tang, Tianhang Su, Fangyuan Hu, Jianning Lu, Youheng Yu, Ziqi Liu, Pinghua Yang, Zhangjun Cheng

**Affiliations:** ^1^ Department of Hepatobiliary and Pancreatic Surgery, Zhongda Hospital, Southeast University, Nanjing, China; ^2^ Department of General Surgery, Jinling Hospital, Affiliated Hospital of Medical School, Nanjing University, Nanjing, China; ^3^ Department of Hepatic Surgery IV, the Eastern Hepatobiliary Surgery Hospital, Naval Medical University, Shanghai, China

**Keywords:** hepatocellular carcinoma (HCC), tumor burden score (TBS), hepatectomy, alphafetoprotein (AFP), estimated hepatectomy extent

## Abstract

**Purpose:**

To develop nomogram models predicting the prognosis for patients with hepatocellular carcinoma (HCC) before hepatectomy.

**Methods:**

Patients treated at the Eastern Hepatobiliary Surgery Hospital and Zhongda Hospital, Southeast University, from January 2012 to July 2014, were retrospectively enrolled. Prediction models for overall survival (OS) and recurrence-free survival (RFS) were constructed.

**Results:**

A total of 1117 patients with HCC were enrolled in this study, and were divided into a training cohort (n=838) and a validation cohort (n=279). A prediction model for OS in the training cohort (OS-nomo, C-index=0.71), including alpha-fetoprotein (AFP), estimated hepatectomy extent, and tumor burden score (TBS) as independent factors (all P<0.05), was constructed. For clinical application, we stratified all patients into three distinct risk groups: low-, medium-, and high-risk group for OS, based on total points (TPs). Patients undergoing major hepatectomy, with AFP>20 ng/mL and high level of TBS had the worst OS.

**Conclusion:**

When selecting patients with HCC for hepatectomy, factors including sex, CPS, AFP level, estimated hepatectomy extent, and TBS should be carefully considered. OS-nomo model could serve as important tool for personalized survival prediction.

## 1 Introduction

Hepatocellular carcinoma (HCC) is the sixth most common malignancy worldwide and the third leading cause of cancer-related mortality, presenting substantial therapeutic challenges due to its heterogeneous biological behavior and highly variable patient outcomes (1–3). While liver transplantation (LT) provides the most favorable oncological results by eradicating both macroscopic and microscopic disease, its application is fundamentally limited by donor organ scarcity and the risk of postoperative complications (4–7). As a result, hepatectomy remains a cornerstone curative intervention, particularly in regions with constrained resources.

According to current guidelines, treatment strategies based on the Barcelona Clinic Liver Cancer (BCLC) staging system recommend hepatectomy predominantly for patients with early-stage, solitary tumors (BCLC-0/A) (8–12). However, this paradigm has been increasingly challenged. Recent multicenter studies have shown that selected patients with multifocal or intermediate-stage HCC (BCLC-B) can derive significant survival benefit from surgical resection, with outcomes superior to those achieved by ablation or transarterial chemoembolization (TACE) in certain subgroups (13–15). For example, a European cohort identified BCLC-B as an independent prognostic factor for resection eligibility, while studies from Australia and Korea reported improved survival with hepatectomy compared to non-surgical modalities (14–16). These findings highlight the urgent need to refine patient selection criteria and to consider extending surgical indications beyond traditional BCLC boundaries.

A major obstacle to expanding the indications for hepatectomy is the lack of robust tools for accurately stratifying patients who are most likely to benefit from surgery. Existing prognostic models often rely on static parameters such as tumor size and number. The tumor burden score (TBS), which integrates tumor size and number into a single geometric metric, provides a more comprehensive and dynamic assessment of disease extent (17–19). Preliminary studies suggest that TBS more accurately predicts post-resection outcomes than conventional staging systems. Nevertheless, its role in guiding preoperative decision-making remains insufficiently explored.

This study addresses these gaps by developing and validating TBS-based nomograms to preoperatively stratify HCC patients. We hypothesize that TBS, combined with clinicopathological variables (e.g., alpha-fetoprotein, hepatectomy extent), will enable personalized risk prediction, thereby optimizing surgical candidate selection and resource allocation.

## 2 Materials and methods

### 2.1 Study cohort

This multicenter retrospective cohort study encompassed a period spanning from January 2012 to July 2014. The period from 2012 to 2014 was selected due to the substantial and concentrated number of HCC patients at Zhongda Hospital, Southeast University, as well as the availability of patient data exported from Eastern Hepatobiliary Surgery Hospital. A total of 1432 patients who underwent hepatectomy for HCC at Eastern Hepatobiliary Surgery Hospital and Zhongda Hospital, Southeast University were retrospectively enrolled through systematic electronic medical record (EMR) review complemented by paper-based operative reports for cross-verification. Furthermore, the time frames for patient inclusion were identical at both hospitals. At diagnosis and surgery, data on patients’ demographic characteristics, disease presentation, liver function condition, the estimated hepatectomy extent, tumor size and number, BCLC stage, and treatments were recorded. Their survival status was recorded every 3 months during the disease course until death or cessation of follow-up.

Patients were followed through September 2019 using a multimodal approach (outpatient review, telephone follow-up and medical record review): 1) quarterly outpatient clinical reviews, 2) structured telephone interviews, and 3) mortality registry cross-checking. The final follow-up completion rate reached 91.4% (1,309/1,432), with 123 cases censored due to either loss to follow-up due to inability to contact patients via telephone or outpatient follow-up (n=89) or voluntary withdrawal (n=34). For incomplete cases, survival time was calculated from surgery date to last verified contact, with censoring status explicitly documented in analysis.

**Figure 1:**
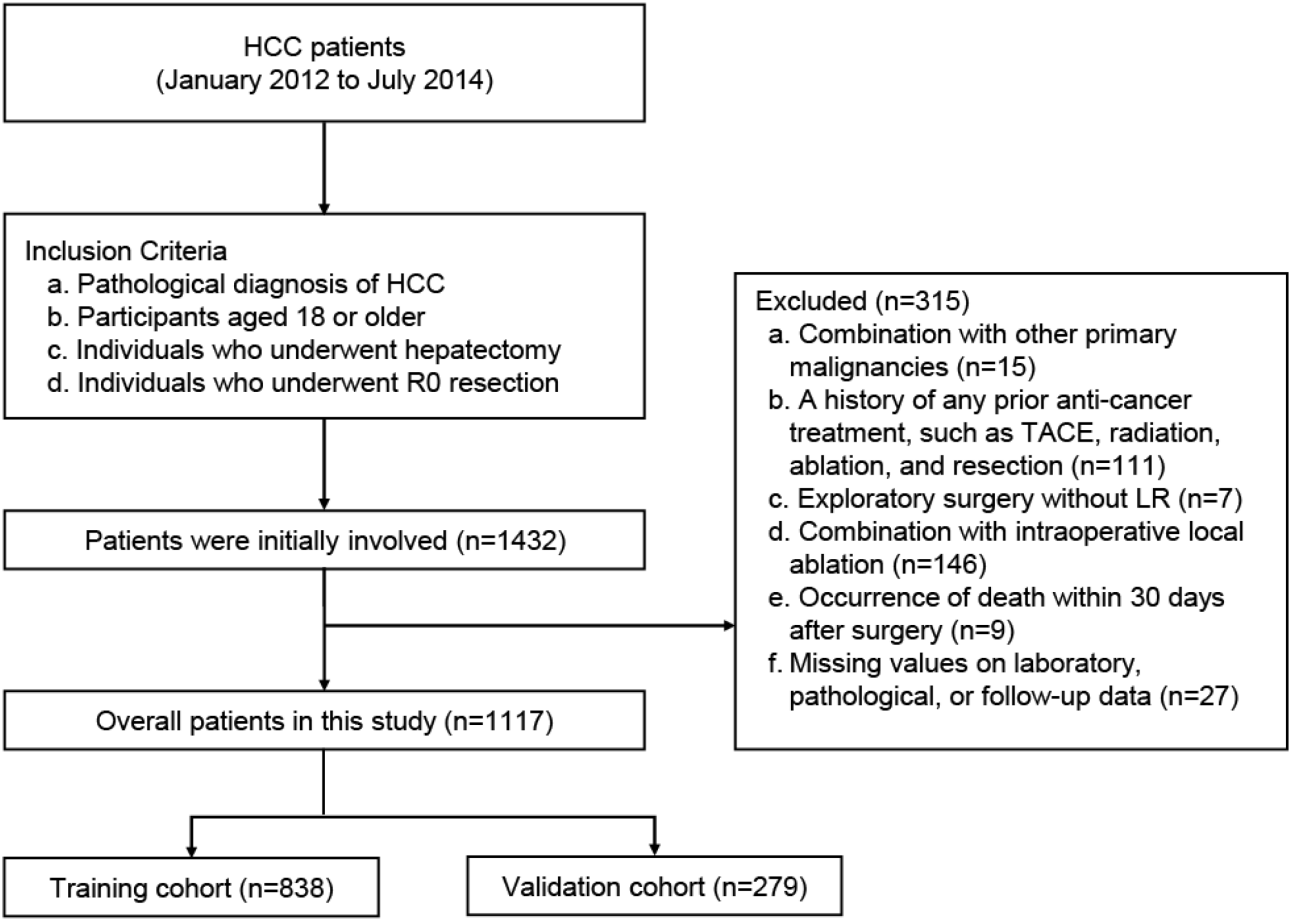
Study profile.

The inclusion criteria were as follows: (a) pathological diagnosis of HCC; (b) age 18 years or older; (c) history of hepatectomy; (d) Individuals who underwent R0 resection. The exclusion criteria were as follows: (a) comorbid with other primary malignancies; (b) a history of any prior anti-cancer treatment, such as TACE, LT, ablation, and resection; (c) exploratory surgery without liver resection; (d) combination with intraoperative local ablation; (e) occurrence of death within 30 days after surgery; or (f) missing values on laboratory, pathological, or follow-up data. The screening process is shown in **Figure 1**. All the included patients were randomly divided into a training cohort (accounting for 75%) and a validation cohort (accounting for 25%).

### 2.2 Patients’ characteristics

Demographic and clinicopathologic data included sex, age, body mass index (BMI), infection of hepatitis B virus (HBV), antiviral therapy, BCLC stage, Child-Pugh score, preoperative ascites or esophageal and gastric varices (EGV), laboratory values [such as platelet count, prothrombin time (PT), international normalized ratio (INR), total bilirubin (TBIL), albumin, and alpha-fetoprotein (AFP)], estimated hepatectomy extent (minor and major), tumor size, and tumor number.

### 2.3 Diagnosis and definition

Patients were categorized as BCLC 0-A stage (single tumor or 2–3 nodules, each ≤3 cm) and BCLC B stage (2–3 nodules, >3 cm or ≥4 nodules), without macrovascular invasion, extrahepatic spread according to imaging findings, and cancer-related symptoms (PS-0).12 R0 resection referred to the complete removal of the tumor, with negative microscopic margins upon microscopic examination. BMI was calculated by dividing the patient’s weight (in kilograms) by height (in meters squared) or BMI = weight (in kg)/height^2^ (in m^2^). Patients were categorized as underweight (BMI<18.5, kg/m^2^), normal (18.5≤BMI<24, kg/m^2^), overweight (24≤BMI<28, kg/m^2^), or obese (BMI≥28, kg/m^2^) (20, 21). AFP was divided into 2 grades: low (≤20 ng/mL) and high (>20 ng/mL) levels, as previously reported (22). Minor liver resections were defined as the resection of 2 segments or less. Child-Pugh Score (CPS) was calculated based on the following five aspects: hepatic encephalopathy, ascites, bilirubin, prothrombin time, and albumin (23, 24).

### 2.4 Calculation of TBS

TBS was defined as the distance from the origin on a Cartesian plane incorporating maximum tumor size (x-axis) and tumor number (y-axis), based on the theory of the Metro ticket paradigm (17–19). Tumor size was measured in centimeters (cm). For patients with multiple tumors, the tumor number was determined according to the total number of lesions identified by preoperative imaging (Computed Tomography or Magnetic Resonance Imaging). TBS was divided into 3 groups: low (TBS<3.36), medium (TBS 3.36-13.74), and high (TBS>13.74) level, calculated based on the Pythagorean theorem: TBS^2^ = (maximum tumor size)^2^ + (tumor number)^2^. The TBS grouping thresholds were based on previously published studies, which demonstrated that this classification provides good prognostic stratification in clinical practice (25–27).

### 2.5 Outcomes and follow-up

The OS was the primary endpoint, defined as the interval between the date of hepatectomy and data of death by any cause or last follow-up. RFS was the second endpoint, defined as the interval between the date of hepatectomy and the date of recurrence of HCC or death or last follow-up with no tumor relapse.

### 2.6 Statistical analysis

Cases were randomly assigned to the training cohort (75%) and validation cohort (25%) using a random sampling method in R software (version 4.2.3). To ensure reproducibility of the results, the random seed was set at 1000.

The normality of continuous variables was evaluated using the Shapiro-Wilk test. Parametric continuous data were expressed as the mean ± standard deviation (SD) and compared using the Independent Samples T-test. While non-parametric data were expressed as median and interquartile ranges (IQR), and compared between the training and validation cohorts using the Mann-Whitney U test. Categorical variables were expressed as numbers and percentages and compared using the χ^2^ test. Survival curves were calculated using the Kaplan-Meier method and compared using the log-rank test.

To determine the independent prognostic factors of OS and RFS, the Cox proportional hazards model was used for univariate and multivariate analysis. A prognostic nomogram model was constructed based on these factors to predict patient outcomes. The bootstrap resampling method was chosen for internal validation of the predictive models, with 1000 repetitions. The concordance index (C-index) and area under the time-dependent receiver operating characteristic curve (AUC-TD) calculated by bootstrapping were used to evaluate discrimination ability in both the training cohort and the validation cohort. The ability of calibration was assessed by the calibration curve, which was evaluated by comparing the nomogram-predicted probability with the actual probability. The clinical utility of the nomogram was further assessed using decision curve analysis (DCA) to evaluate its net benefit across different probability thresholds. For clinical application of the model, the total scores of each patient were calculated based on the nomogram. The optimal cut-off point for the scores was selected using X-tile (28). The C-index, AUC-TD, and the net reclassification index (NRI) were used to evaluate the clinical benefits and utility of the nomogram compared with the BCLC stage.

In univariate analysis, P<0.05 was considered to indicate statistical significance, while in other analyses, P<0.05 was considered as indicative of significance. Statistical analysis was performed by using R software (version 4.2.3), IBM SPSS Statistics (version 27), and X-tile (version 3.6.1).

## 3 Results

### 3.1 Characteristics of the study cohort

During the study period, a total of 1117 patients underwent hepatectomy and were included in the final study cohort, and the patients were randomly divided into the training cohort (n=838) and validation cohort (n=279) (**Figure 1**). The baseline characteristics are listed in **Table 1**, **S1**. Among these patients in the overall cohort, most were male (n=938, 85%). The majority of patients (n=1023, 91.6%) were in BCLC 0-A stage, while the others (n=94, 8.4%) were in BCLC B stage. Further, 79.1% of patients (n=884) underwent minor hepatectomy; 61.7% of patients (n=689) were in a low level of preoperative AFP (AFP ≤ 20 ng/mL); while 27.3% of patients (n=305) were at a low level of TBS, 66.1% (n=738) at medium level, and 6.6% (n=74) at high level.

**Table 1:** Baseline characteristics.

Characteristic	Overall (n = 1117^A^)	Training cohort (n = 838^A^)	Validation cohort (n = 279^A^)	P
**Sex**				0.808
Female	179 (16.0)	133 (15.9)	46 (16.5)	
Male	938 (84.0)	705 (84.1)	233 (83.5)	
**Age**, years	54 (46–61)	53 (46–61)	57 (49–64)	< 0.001
**BMI**				0.777
Underweight	41 (3.7)	31 (3.7)	10 (3.6)	
Normal	632 (56.6)	472 (56.3)	160 (57.3)	
Overweight	357 (31.9)	273 (32.6)	84 (30.1)	
Obesity	87 (7.8)	62 (7.4)	25 (9.0)	
**Positive HBsAg**	938 (84.0)	713 (85.1)	225 (80.6)	0.080
**Antiviral therapy**	181 (16.2)	131 (15.6)	50 (17.9)	0.369
**BCLC stage**				0.537
0-A	1023 (91.6)	765 (91.3)	258 (92.5)	
B	94 (8.4)	73 (8.7)	21 (7.5)	
**CPS**				0.274
A5	993 (88.9)	750 (89.5)	243 (87.1)	
A6	109 (9.7)	77 (9.2)	32 (11.5)	
B7	13 (1.2)	9 (1.1)	4 (1.4)	
B8	2 (0.2)	2 (0.2)	0 (0.0)	
**Preoperative ascites**	64 (5.7)	48 (5.7)	16 (5.7)	0.997
**EGV**	286 (25.6)	213 (25.4)	73 (26.2)	0.804
**Preoperative platelets**, 10^9^/L	160 (116–202)	161 (118–203)	160 (107–200)	0.367
**Preoperative PT**, seconds	12.3 (11.0–14.8)	12.3 (11.1–14.8)	12.5 (10.6-15.0)	0.979
**Preoperative INR**	0.99 (0.95–1.04)	0.99 (0.95-1.04)	0.99 (0.94-1.05)	0.698
**Preoperative TBIL**, μmol/L	13.5 (10.6-16.8)	13.8 (10.6-17.1)	13.1 (10.3-16.4)	0.081
**Preoperative albumin**, g/L	41.6 (38.9–44.3)	41.5 (38.9-44.3)	41.7 (38.6-44.3)	0.772
**Preoperative AFP**, ng/mL				0.582
≤ 20	413 (37.0)	306 (36.5)	107 (38.4)	
> 20	704 (63.0)	532 (63.5)	172 (61.6)	
**Estimated hepatectomy extent**				0.475
Minor	884 (79.1)	659 (78.6)	225 (80.6)	
Major	233 (20.9)	179 (21.4)	54 (19.4)	
**Tumor size**, cm	4.7 (3.1–8.0)	4.8 (3.1-8.2)	4.6 (3.1-7.0)	0.401
**Tumor number**				0.404
Solitary	998 (89.3)	745 (88.9)	253 (90.7)	
Multiple	119 (10.7)	93 (11.1)	26 (9.3)	
**TBS**				0.198
Low	305 (27.3)	219 (26.1)	86 (30.8)	
Medium	738 (66.1)	566 (67.5)	172 (61.6)	
High	74 (6.6)	53 (6.3)	21 (7.5)	

^A^n (%); Median (interquartile range, IQR)

BCLC, Barcelona Clinic Liver Cancer; CPS, Child-Pugh score; EGV, esophageal and gastric varices; PT, prothrombin time; INR, international normalized ratio; TBIL, total bilirubin; ALB, albumin; AFP, alpha fetoprotein; TBS, tumor burden score; IQR, interquartile range.

### 3.2 Prediction models for overall survival

**Figure 2:**
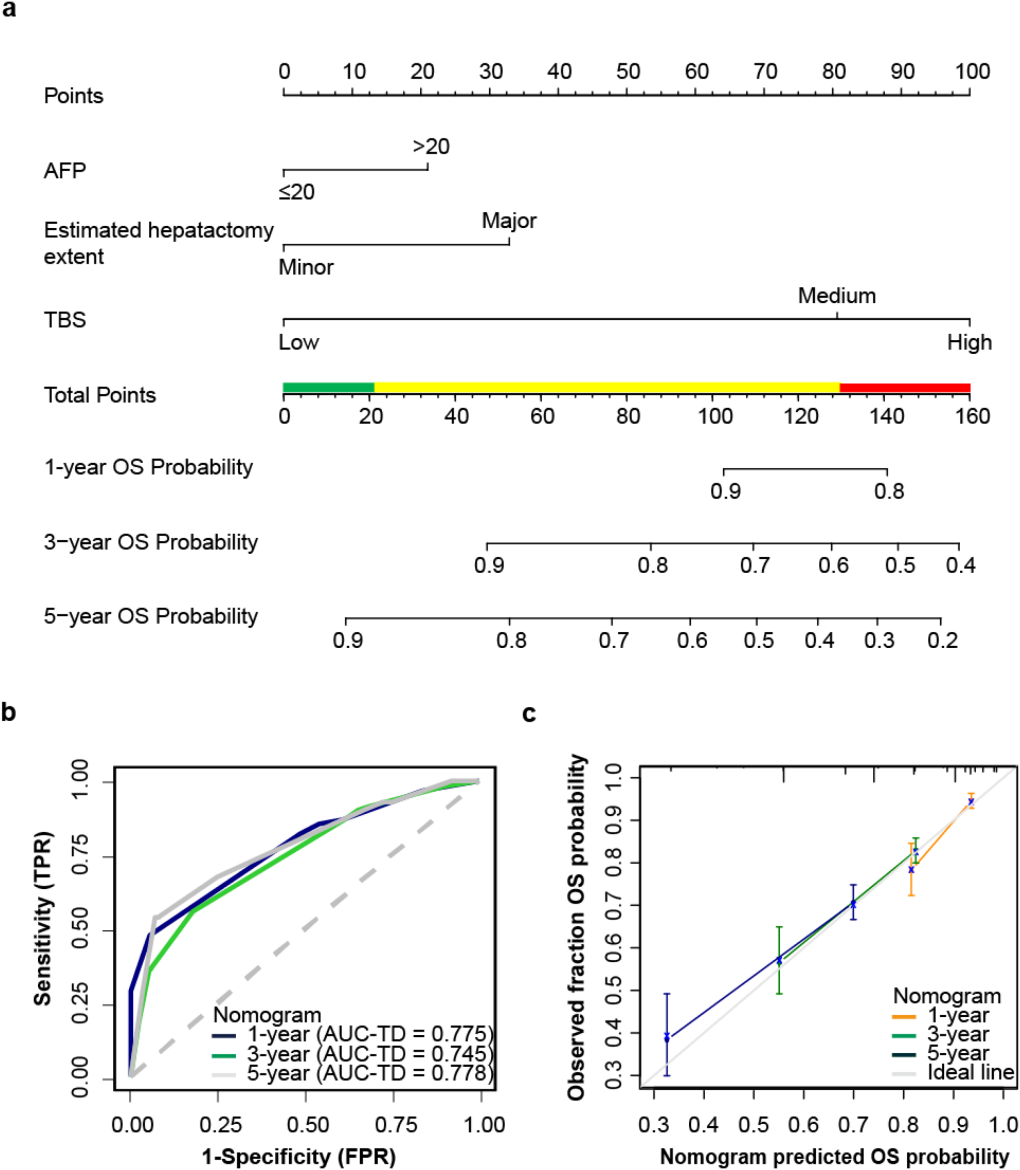
Nomogram for OS. **(a)** The nomogram maps the predicted probability of 1-, 3-and 5-years OS on a scale of 0 to 160. For each covariate, a vertical line is drawn upwards and the corresponding points (such as high level of TBS = 100 points) are noted. This is repeated for each covariate to obtain a total score that corresponds to a predicted probability of 1-, 3- and 5-year OS at the bottom of the nomogram. Total points were divided into 3 groups according to X-tile, low-risk group (green band), medium-risk group (yellow band), and high-risk group (red band). **(b)** Time-dependent receiver operating characteristic curves for 1-, 3- and 5-year OS. **(c)** Calibration curves for 1-, 3- and 5-year OS probability.

**Figure 3:**
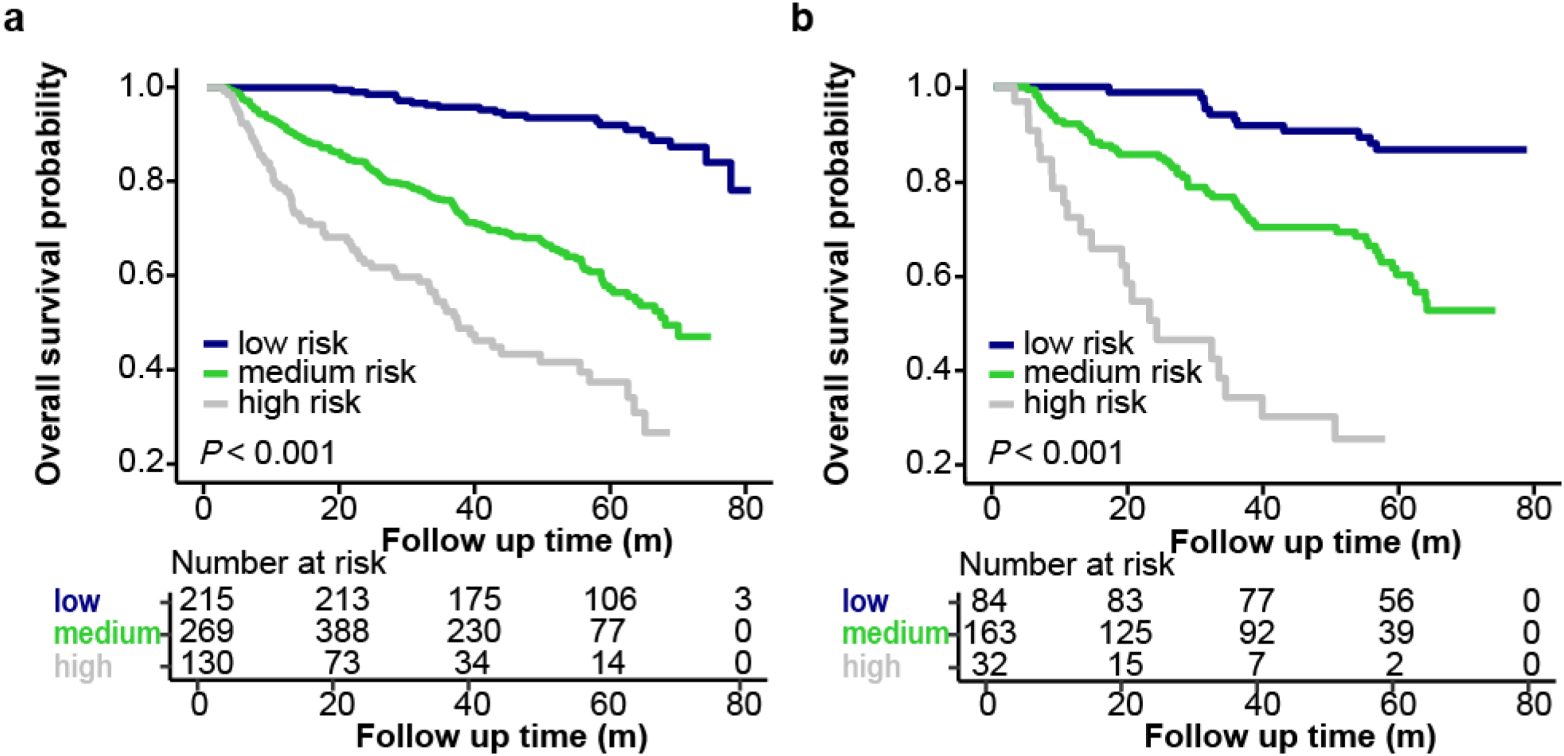
Kaplan-Meier curve demonstrating differences in OS between patients with low, median or high death risk. **(a)** The training cohort. **(b)** The validation cohort.

In the overall cohort, a total of 357 patients (32.0%) died during the follow-up period. The rates of survival at 1, 3, and 5 years were 91.4%, 77%, and 64.8%, respectively. In the training cohort, based on univariate Cox analysis of OS, antiviral therapy (P<0.001), BCLC stage (P<0.001), CPS (P=0.001), preoperative ascites (P=0.045), preoperative INR (P=0.004), preoperative TBIL (P=0.003), preoperative AFP (P<0.001), estimated hepatectomy extent (P<0.001), tumor size (P<0.001), tumor number (P=0.01) and TBS (P<0.001) were identified as risk factors (**Table 2**). Furthermore, a multivariable Cox analysis based on these risk factors confirmed that AFP, estimated hepatectomy extent and TBS were independent risk factors for OS (**Supplementary Figure S1**). The results were utilized to develop a nomogram called OS-nomo for prediction of 1-, 3-, 5-year OS (**Figure 2a**). The nomogram revealed that patients who expected to undergo major hepatectomy, had AFP>20 ng/mL, and with high level of TBS had the poorest OS after hepatectomy. OS-nomo demonstrated good predictive ability, with a C-index of 0.71 (95%CI, 0.696–0.724). Application of the nomogram yielded an AUC-TD of 0.775 (95%CI, 0.733–0.818), 0.745 (95%CI, 0.708–0.783) and 0.778 (95%CI, 0.734–0.821) for 1-, 3-, 5-year OS in the training cohort, respectively (**Figure 2b**). While in the validation cohort, the OS-nomo yielded an AUC-TD of 0.757 (95%CI, 0.683-0.831), 0.725 (95%CI, 0.663-0.787) and 0.758 (95%CI, 0.697-0.82) for 1-, 3-, 5-year OS, respectively. The calibration curve plots for predicting 1-, 3-, and 5-year OS are shown in **Figure 2c**, where the points only slightly deviated from the 45-degree line, indicating a high goodness of fit between predicted and observed values. The X-tile program was used to determine the cut-off values for total points (TPs), which were subsequently utilized to stratify patients into three risk groups based on OS. Patients were classified into the following groups: low death-risk group (TPs ≤ 21, n=215), medium death-risk group (TPs: 21-132.9, n=493), and high death-risk group (TPs>132.9, n=130). The Kaplan-Meier OS curves of this nomogram for OS showed significant discrimination between the three risk groups in both the training cohort and the validation cohort (P<0.001, **Figure 3**). The probability of 1-, 3-, 5-year OS for patients in the low death-risk group are 100%, 95.8%, and 91.9%, respectively. For patients in the median death-risk group, the corresponding values are 90.8%, 75.7%, and 56.5%, respectively; while for patients in the high death-risk group, they are 77.6%, 51.7%, and 36.5%, respectively.

**Table 2:** Univariate analysis for OS and RFS in the training cohort.

Characteristic	Overall Survival		Recurrence Free Survival	
	HR	P-value	HR	P-value
**Sex**				
Female	Ref		Ref	
Male	1.1 (0.78-1.54)	0.594	1.72 (1.27-2.32)	< 0.001
**Age**, years	1.01 (1-1.02)	0.177	1 (0.99–1.01)	0.452
**BMI**				
Underweight	Ref		Ref	
Normal	1 (0.47-2.15)	0.985	1.08 (0.59-1.94)	0.820
Overweight	1.11 (0.7-1.77)	0.663	1.04 (0.73-1.49)	0.822
Obese	0.78 (0.48-1.29)	0.333	0.82 (0.56-1.19)	0.291
**Positive HBsAg**	0.89 (0.65-1.23)	0.477	1.3 (0.98-1.72)	0.067
**Antiviral therapy**	0.49 (0.33-0.73)	< 0.001	0.81 (0.62-1.07)	0.134
**BCLC stage**				
0-A	Ref		Ref	
B	2.03 (1.41-2.91)	< 0.001	1.48 (1.08-2.02)	0.016
**CPS**	1.44 (1.1-1.88)	0.001	1.43 (1.14-1. 78)	0.002
**Preoperative ascites**	1.66 (1.01-2.71)	0.045	2.19 (1.53-3.12)	< 0.001
**EGV**	1.23 (0.94-1.6)	0.131	1.15 (0.93-1.42)	0.206
**Preoperative platelets**, 10^9^/L	1 (1–1)	0.144	1 (1–1)	0.490
**Preoperative PT**, seconds	1 (0.97-1.03)	0.999	1 (0.98-1.02)	0.987
**Preoperative INR**	8.91 (2-39.7)	0.004	6.08 (1.88-19.7)	0.003
**Preoperative TBIL**, μmol/L	1.04 (1.01-1.06)	0.003	1.01 (0.99-1.03)	0.191
**Preoperative ALB**, g/L	0.99 (0.96-1.01)	0.321	0.97 (0.95-0.99)	0.009
**Preoperative AFP**, ng/mL				
≤ 20	Ref		Ref	
> 20	1.85 (1.42-2.43)	< 0.001	1. 58 (1. 29-1.94)	< 0.001
**Estimated hepatectomy extent**				
Minor	Ref		Ref	
Major	3.13 (2.42-4.04)	< 0.001	1.99 (1. 6-2.46)	< 0.001
**Tumor size**, cm	1.13 (1.1-1.16)	< 0.001	1.09 (1.07-1.12)	< 0.001
**Tumor number**				
Solitary	Ref		Ref	
Multiple	1.57 (1.11-2.21)	0.010	1.25 (0.94-1.67)	0.131
**TBS**				
Low	Ref		Ref	
Medium	0.07 (0.04–0.13)	< 0.001	0.21 (0.14-0.31)	< 0.001
High	0.42 (0.28–0.62)	< 0.001	0.51 (0.36–0.72)	< 0.001

HR, hazard ratio; CI, confidence interval; Ref, reference.

To compare the accuracy between the nomogram and the BCLC stage, C-index, AUC-TD, and NRI were calculated, as shown in **Table 3**. The C-index of the nomogram was 0.71 (95%CI, 0.696–0.724), while that for the BCLC stage was 0.534 (95%CI, 0.523–0.545). The DCA demonstrated favorable clinical utility of the model, with net benefit values exceeding default strategies (treat-all or treat-none or BCLC stage) across a wide range of threshold probabilities. The NRI of 1, 3, 5-year OS compared with the BCLC stage was 0.129, 0.347, and 0.393, respectively.

**Table 3:** C-index, AUC, and NRI of OS-nomo, BCLC stage, tumor size and tumor number in survival prediction.

Evaluation metrics	OS-nomo		BCLC stage	Tumor size	Tumor number
	Training cohort	Validation cohort	Training cohort		
**C-index**	0.71 (0.696–0.724)	0.691 (0.668-0.714)	0.534 (0.523–0.545)	0.698 (0.682-0.714)	0.527 (0.515-0.539)
**AUC-TD**					
For 1-year OS	0.775 (0.733–0.818)	0.757 (0.683-0.831)	0.549 (0.504–0.593)	0.772 (0.725-0.82)	0.543 (0.497-0.589)
For 3-year OS	0.745 (0.708–0.783)	0.725 (0.663-0.787)	0.539 (0.512–0.567)	0.725 (0.685-0.766)	0.534 (0.505-0.564)
For 5-year OS	0.778 (0.734–0.821)	0.758 (0.697-0.82)	0.549 (0.522–0.575)	0.765 (0.72-0.811)	0.533 (0.504-0.563)
**NRI (vs. BCLC stage)**					
For 1-year OS	0.129 (0- 0.366)				
For 3-year OS	0.347 (0.213- 0.405)				
For 5-year OS	0.393 (0.254- 0.473)				

### 3.3 Prediction models for recurrence-free survival

During the study period, 51.7% patients underwent recurrence of HCC. The 1-, 3-, 5-year RFS rates were 68.2%, 53.4%, and 45% respectively. According to the univariate Cox regression in the training cohort, sex (P<0.001), BCLC stage (P=0.016), CPS (P=0.002), preoperative ascites (P<0.001), preoperative INR (P=0.003), preoperative ALB (P=0.009), preoperative AFP (P<0.001), estimated hepatectomy extent (P<0.001), tumor size (P<0.001), and TBS (P<0.001) may be risk factors, as shown in **Table 2**. Furthermore, a multivariable Cox analysis based on these risk factors confirmed that sex, CPS, AFP, estimated hepatectomy extent, and TBS were independent risk factors for RFS (**Supplementary Figure S3**). A nomogram for prediction of RFS probability based on the results was established (**Supplementary Figure S4a**). The RFS nomogram model demonstrated moderate prediction capability, as indicated by a C-index of 0.654 (95%CI, 0.641–0.667). Using the same method, we used X-tile to stratify patients into three risk groups based on total points (TP). The three risk categories were as follows: low recurrence-risk group (TP ≤ 104, n=284), medium recurrence-risk group (TP: 104-164, n=419), and high recurrence-risk group (TP>164, n=135). The Kaplan-Meier RFS curves of TP showed significant discrimination between the three risk groups (P<0.001, **Supplementary Figure S5**).

## 4 Discussion

In this retrospective study, AFP, estimated hepatectomy extent, and TBS were identified as independent predictors of OS in HCC patients undergoing hepatectomy. Based on these variables, nomogram models were developed and validated to predict OS and RFS at 1, 3, and 5 years. Patients were stratified into low-, medium-, and high-risk groups for both OS and RFS using these nomograms, which can assist clinicians in individualized risk assessment and decision-making regarding hepatectomy.

The findings are consistent with previous studies that have established AFP and TBS as significant prognostic markers in HCC patients after hepatectomy (25, 26, 29, 30). The prognostic value of TBS, in particular, has been widely validated and was confirmed as the most significant variable in our OS nomogram (26, 27). Its prognostic predictive efficacy was significantly superior to that of tumor size and number alone. Finally, OS-nomo and RFS-nomo include the variable of estimated hepatectomy extent. Minor hepatectomy may involve the removal of smaller portions of liver tissue, which results in minimal impairment of liver function. Compared to prior models, OS-nomo and RFS-nomo integrate these key factors, increase the sample size and provide a user-friendly tool for clinical application.

Notably, the RFS nomogram suggests that sex may play a role in recurrence risk, although the underlying mechanisms remain unclear. While some studies have reported sex-related differences in HCC prognosis, the evidence is still inconclusive and warrants further investigation (31–33). The results contribute to this ongoing discussion but should be interpreted with caution.

The CPS demonstrated no statistical significance in the OS-nomog but retained prognostic value in the RFS-nomo. This phenomenon may be attributed to the fact that the enrolled HCC cohort predominantly comprised Child-Pugh A patients, indicating well-compensated hepatic function across the study population. Within this relatively homogeneous low-risk cohort, the CPS lacks sufficient discriminatory power for long-term mortality risk stratification. Conversely, RFS, as a more sensitive early-phase prognostic indicator, remains capable of detecting subtle variations in hepatic functional reserve that may influence the tumor recurrence microenvironment through mechanisms such as altered immune surveillance, metabolic reprogramming, and peritumoral stromal remodeling.

The nomogram models developed in this study incorporate multiple variables such as AFP, estimated hepatectomy extent, and TBS. While these variables enhance the predictive accuracy, they also increase the complexity of the model. Overfitting can occur if the model is too complex relative to the amount of data available, leading to excellent performance on the training data but poor generalizability to new, unseen data. Therefore, we employed bootstrap resampling and internal validation to mitigate the risk of overfitting.

This study further confirms the predictive ability of TBS in assessing the prognosis of patients with HCC undergoing hepatectomy. The nomogram models in this study can be useful to surgeons and hepatologists by enabling them to quickly calculate the surgical risk to patients and provide personalized guidance for selection of appropriate treatment by risk stratification thresholds and integration into multidisciplinary discussions. However, there were still some limitations in our study. First, the location of the tumor, which may determine the type of liver resection, has not been taken into consideration. Second, these two nomogram models require further validation with more recent multicenter data. Third, overfitting is another potential issue, although rigorous validation procedures were employed to mitigate this risk. Fourth, this study is specifically applicable to open surgery patients and does not encompass those undergoing laparoscopic or robotic-assisted procedures, thereby limiting its generalizability to contemporary minimally invasive surgical approaches. Additionally, excluding patients who had previously received treatment (e.g. TACE, ablation) may introduce selection bias, as the cohort is skewed toward treatment-naive patients. Future studies should systematically analyze these limitations and explore strategies to enhance model robustness.

## 5 Conclusion

In conclusion, the study utilized a large retrospective cohort of patients who had undergone hepatectomy to identify independent factors of OS and RFS. The OS-nomo, which incorporated AFP, estimated hepatectomy extent and TBS, was subjected to internal and external validation and showed good discrimination and calibration. Through the OS-nomo, patients can be classified into three groups: low-death risk, medium-death risk, and high death-risk groups. Additionally, through the RFS-nomo, patients could be classified into three groups: low-recurrence risk, medium-recurrence risk, and high-recurrence risk groups. This indicator could help identify patients who would benefit from surgical resection, thereby optimizing the allocation of scarce liver organ resources.

## Data Availability

The raw data supporting the conclusions of this article will be made available by the authors, without undue reservation.
