# Supplementary material for: Nomogram based on tumor burden score for prediction of prognosis of patients with hepatocellular carcinoma before hepatectomy
Source: Front Oncol. 2025 Jul 8;15:1578859. doi: 10.3389/fonc.2025.1578859 (PMC12279504; doi:10.3389/fonc.2025.1578859)
Supplement: Supplementary file 1 [file DataSheet1.docx]

Supplementary Material

# Supplementary Figures


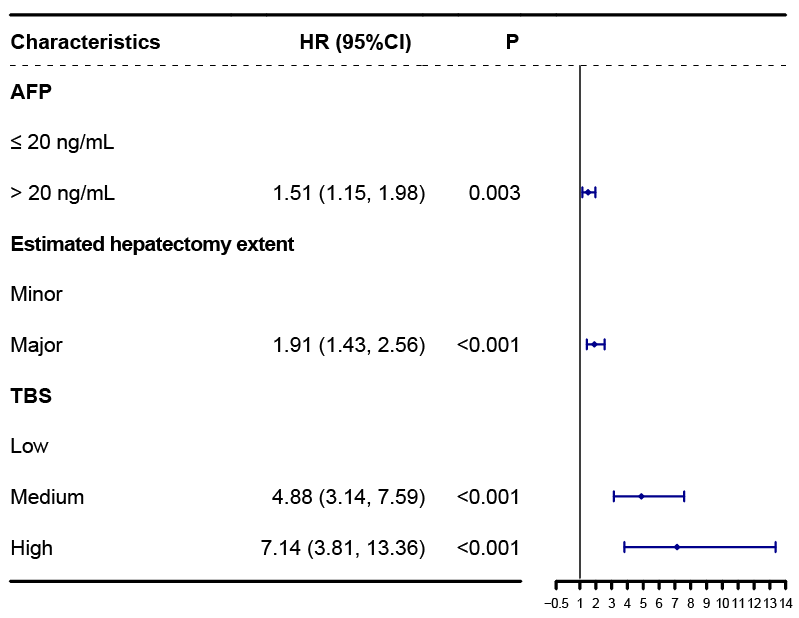


**Figure S1** Forest plot for predicting postoperative OS. Blue dots represent the hazard ratio values, while the error bars represent the 95% confidence intervals.


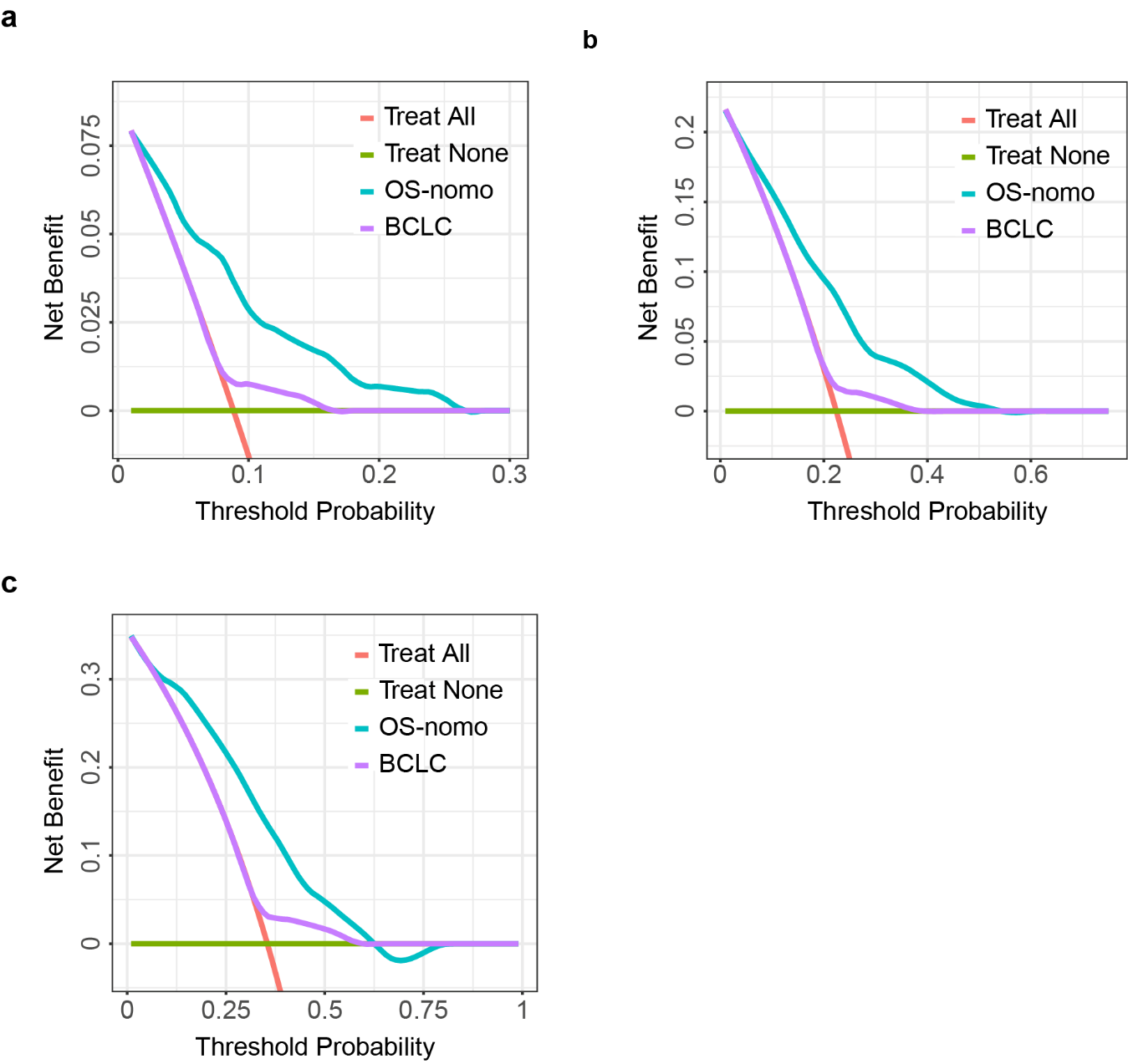


**Figure S2** DCA comparing the OS-nomo and BCLC Stage for predicting OS. (**a**) 1-year OS. (**b**) 3-year OS. (**c**) 5-year OS.


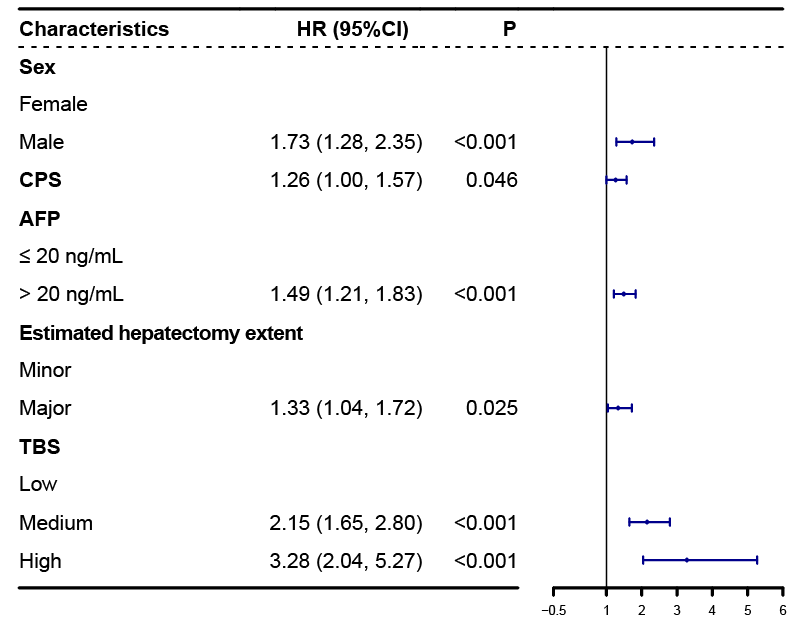


**Figure S3** Forest plot for predicting postoperative RFS. Blue dots represent the hazard ratio values, while the error bars represent the 95% confidence intervals.


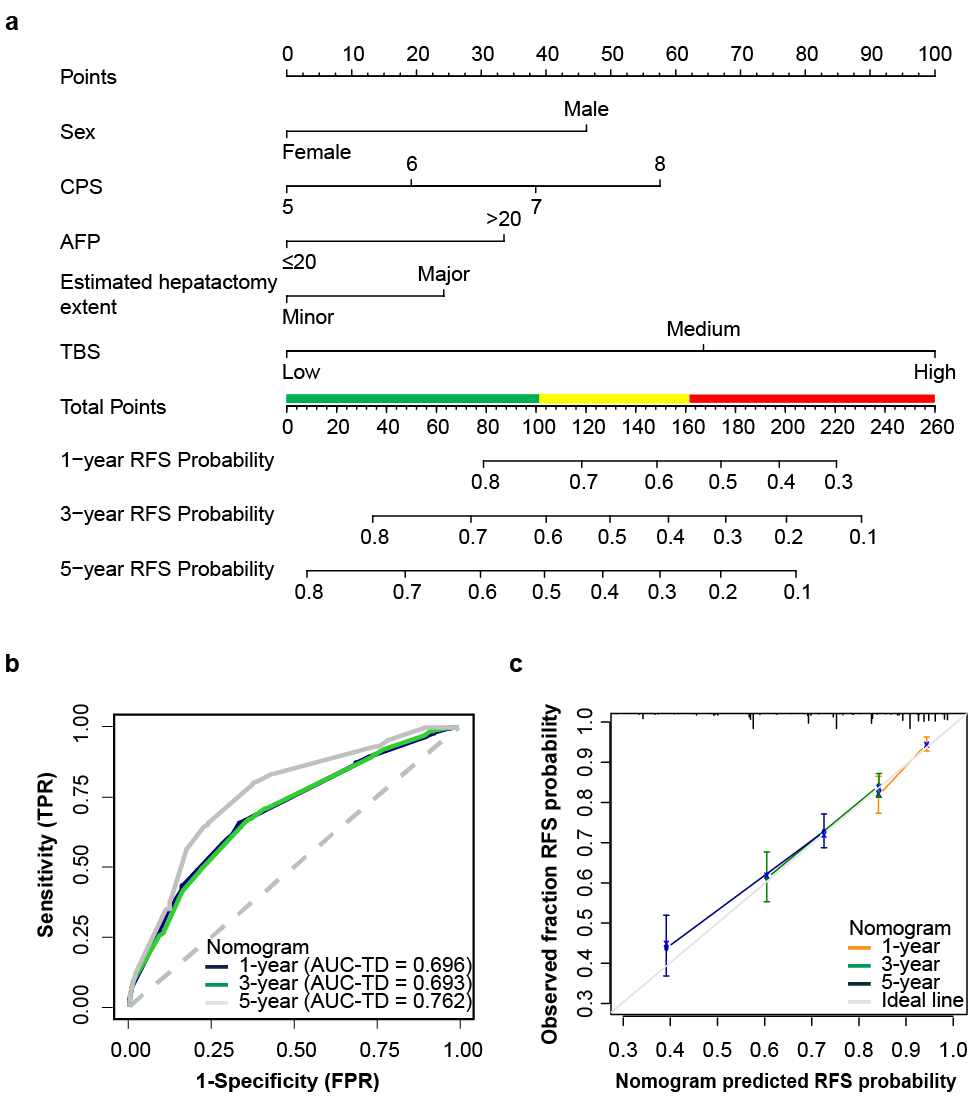


**Figure S4** Nomogram for RFS. (**a**) The nomogram maps the predicted probability of 1-, 3-and 5-year RFS on a scale of 0 to 260. For each covariate, a vertical line is drawn upwards and the corresponding points (such as high level of TBS = 100 points) are noted. This is repeated for each covariate, ending with a total score that corresponds to a predicted probability of 1-, 3- and 5-year RFS at the bottom of the nomogram. (**b**) Time-dependent receiver operating characteristic curves predicting 1-, 3- and 5-year RFS. (**c**) Calibration curves for predicting 1-, 3- and 5-year RFS probability by the nomogram.


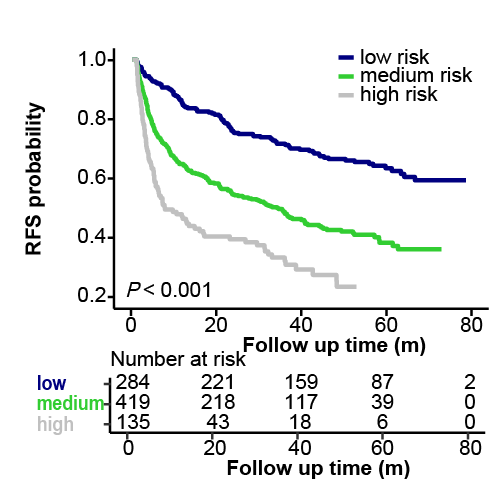


**Figure S5** Kaplan-Meier curve showing differences in RFS between patients with low, median, or high recurrence risk.

# Supplementary Table

**Table S1** Baseline Characteristics of Patients from the Two Hospitals.

| Characteristic | Overall  (n = 1117^A^) | Eastern Hepatobiliary Surgery Hospital  (n = 1053^A^) | Zhongda Hospital  (n = 64^A^) |
| --- | --- | --- | --- |
| Sex |  |  |  |
| Female | 179 (16.0) | 169 (16) | 10 (15.6) |
| Male | 938 (84.0) | 884 (84) | 54 (84.4) |
| Age, years | 54 (46–61) | 54 (46–62) | 51 (41–58) |
| BMI |  |  |  |
| Underweight | 41 (3.7) | 39 (3.7) | 2 (3.1) |
| Normal | 632 (56.6) | 594 (56.4) | 38 (59.4) |
| Overweight | 357 (31.9) | 338 (32.1) | 19 (39.7) |
| Obesity | 87 (7.8) | 82 (7.8) | 5 (7.8) |
| Positive HBsAg | 938 (84.0) | 925 (87.8) | 57 (90.5) |
| Antiviral therapy | 181 (16.2) | 173 (16.4) | 8 (12.5) |
| BCLC stage |  |  |  |
| 0-A | 1023 (91.6) | 963 (91.5) | 60 (93.8) |
| B | 94 (8.4) | 90 (8.5) | 4 (6.2) |
| CPS |  |  |  |
| A5 | 993 (88.9) | 936 (88.9) | 57 (89.1) |
| A6 | 109 (9.7) | 102 (9.7) | 7 (10.9) |
| B7 | 13 (1.2) | 13 (1.2) | 0 (0) |
| B8 | 2 (0.2) | 2 (0.2) | 0 (0) |
| Preoperative ascites | 64 (5.7) | 62 (5.9) | 2 (3.1) |
| EGV | 286 (25.6) | 265 (25.2) | 21 (32.8) |
| Preoperative platelets, 10^9^/L | 160 (116–202) | 160 (115–203) | 161 (119–201) |
| Preoperative PT, seconds | 12.3 (11.0–14.8) | 12.3 (11.1–14.8) | 12.1 (10.8-15.6) |
| Preoperative INR | 0.99 (0.95–1.04) | 0.99 (0.95-1.04) | 0.98 (0.94-1.06) |
| Preoperative TBIL, μmol/L | 13.5 (10.6-16.8) | 13.5 (10.6-16.8) | 13.6 (10.4-16.8) |
| Preoperative albumin, g/L | 41.6 (38.9–44.3) | 41.6 (38.9-44.3) | 41.7 (38.5-44.2) |
| Preoperative AFP, ng/mL |  |  |  |
| ≤ 20 | 413 (37.0) | 395 (37.5) | 18 (28.1) |
| > 20 | 704 (63.0) | 658 (62.5) | 46 (71.9) |
| Estimated hepatectomy extent |  |  |  |
| Minor | 884 (79.1) | 825 (78.3) | 59 (92.2) |
| Major | 233 (20.9) | 228 (21.7) | 5 (7.8) |
| Tumor size, cm | 4.7 (3.1–8.0) | 4.7 (3.1-8) | 5 (3.2-7.6) |
| Tumor number |  |  |  |
| Solitary | 998 (89.3) | 938 (89.1) | 60 (93.8) |
| Multiple | 119 (10.7) | 115 (10.9) | 4 (6.2) |
| TBS |  |  |  |
| Low | 305 (27.3) | 289 (27.4) | 16 (25) |
| Medium | 738 (66.1) | 692 (65.7) | 46 (71.9) |
| High | 74 (6.6) | 72 (6.9) | 2 (3.1) |

**Notes:** ^A^n (%); Median (interquartile range, IQR)

**Abbreviations:** BCLC, Barcelona Clinic Liver Cancer; CPS, Child-Pugh score; EGV, esophageal and gastric varices; PT, prothrombin time; INR, international normalized ratio; TBIL, total bilirubin; ALB, albumin; AFP, alpha fetoprotein; TBS, tumor burden score; IQR, interquartile range.
